# Robotic versus open pancreatoduodenectomy after completion of the learning curve: a propensity score–matched and CUSUM analyses of perioperative outcomes

**DOI:** 10.1007/s00464-026-13143-3

**Published:** 2026-07-23

**Authors:** Matteo Aulicino, Giuseppe Quero, Claudio Fiorillo, Roberta Menghi, Giuseppe Daloiso, Flavia Taglioni, Claudia Ferrantone, Davide De Sio, Antonio Pio Tortorelli, Vincenzo Tondolo, Sergio Alfieri

**Affiliations:** 1 Pancreatic Surgery Unit, Fondazione Policlinico Agostino Gemelli IRCCS of Rome, Rome, Italy; 2 Dipartimento di Medicina e Chirurgia Traslazionale, Università Cattolica del Sacro Cuore di Roma, Rome, Italy; 3 General Surgery, Department of Medical and Surgical Sciences, Fondazione Policlinico Universitario Agostino Gemelli IRCCS, Rome, Italy; 4 Digestive Surgery Unit, Department of Translational Medicine, Catholic University of the Sacred Heart, Rome, Italy

**Keywords:** Robot-assisted pancreaticoduodenectomy, Pancreaticoduodenectomy, CUSUM analysis, Perioperative outcomes, Learning curve

## Abstract

**Background:**

Robotic pancreatoduodenectomy (R-PD) has emerged as a minimally invasive alternative to open pancreatoduodenectomy (O-PD), although its role remains debated due to technical complexity and heterogeneous outcomes. This study compared perioperative outcomes between R-PD and O-PD in a high-volume center while minimizing the impact of the learning curve, and additionally evaluated the progressive improvement in surgical outcomes between the early and late phases of the robotic experience through comparative analyses and CUSUM assessment.

**Methods:**

A single-center propensity score–matched retrospective cohort study was conducted using a prospectively maintained database of patients undergoing PD between January 2020 and December 2025. The most recent 50 consecutive R-PDs (late group, LR-PD) were compared with O-PD after 2:1 matching (matched open group, MO-PD) to balance baseline characteristics. Perioperative, postoperative, and pathological outcomes were compared, and multivariable analyses were performed to identify independent predictors of key outcomes. A subgroup analysis comparing the first 50 R-PDs (early group, ER-PD) with the subsequent 50 cases (LR-PD) was also performed, along with CUSUM analyses of outcomes showing differences between the two phases of the robotic experience.

**Results:**

After matching, 100 MO-PD patients were compared with 50 LR-PD patients. LR-PD was associated with significantly lower estimated blood loss (EBL) (200 vs 300 mL; *p* = 0.0012) and shorter length of hospital stay (LOS) (12 vs 14 days; *p* = 0.038), but longer operative time (OT) (582 vs 362 minutes; *p* < 0.001). Rates of major complications (Clavien–Dindo ≥ III) and pancreas-specific complications were comparable between groups. A significantly lower incidence of postoperative lymphatic fistula was observed in the LR-PD (4 vs 15%; *p* = 0.045). At multivariable analysis, the LR-PD was independently associated with lower EBL (*β* − 116.80; *p* = 0.023), shorter LOS (*β* − 1.48; *p* = 0.041), and a reduced risk of postoperative lymphatic fistula (OR 0.30; *p* = 0.047), while remaining an independent predictor of longer OT (*β* 99.75; *p* < 0.001). Within the R-PD, OT was significantly shorter in the LR-PD compared with the ER-PD (582 vs 705 minutes; *p* < 0.001). CUSUM analyses identified inflection points at cases 51, 55, and 53 for OT, major complications, and clinically relevant PPH, respectively.

**Conclusions:**

In a high-volume setting after the learning curve, R-PD is a potentially safe and feasible alternative to O-PD, offering reduced EBL, shorter LOS, and lower incidence of lymphatic fistula, despite longer OT. Further prospective studies are warranted to confirm these findings.

**Graphical abstract:**

**Figure:**
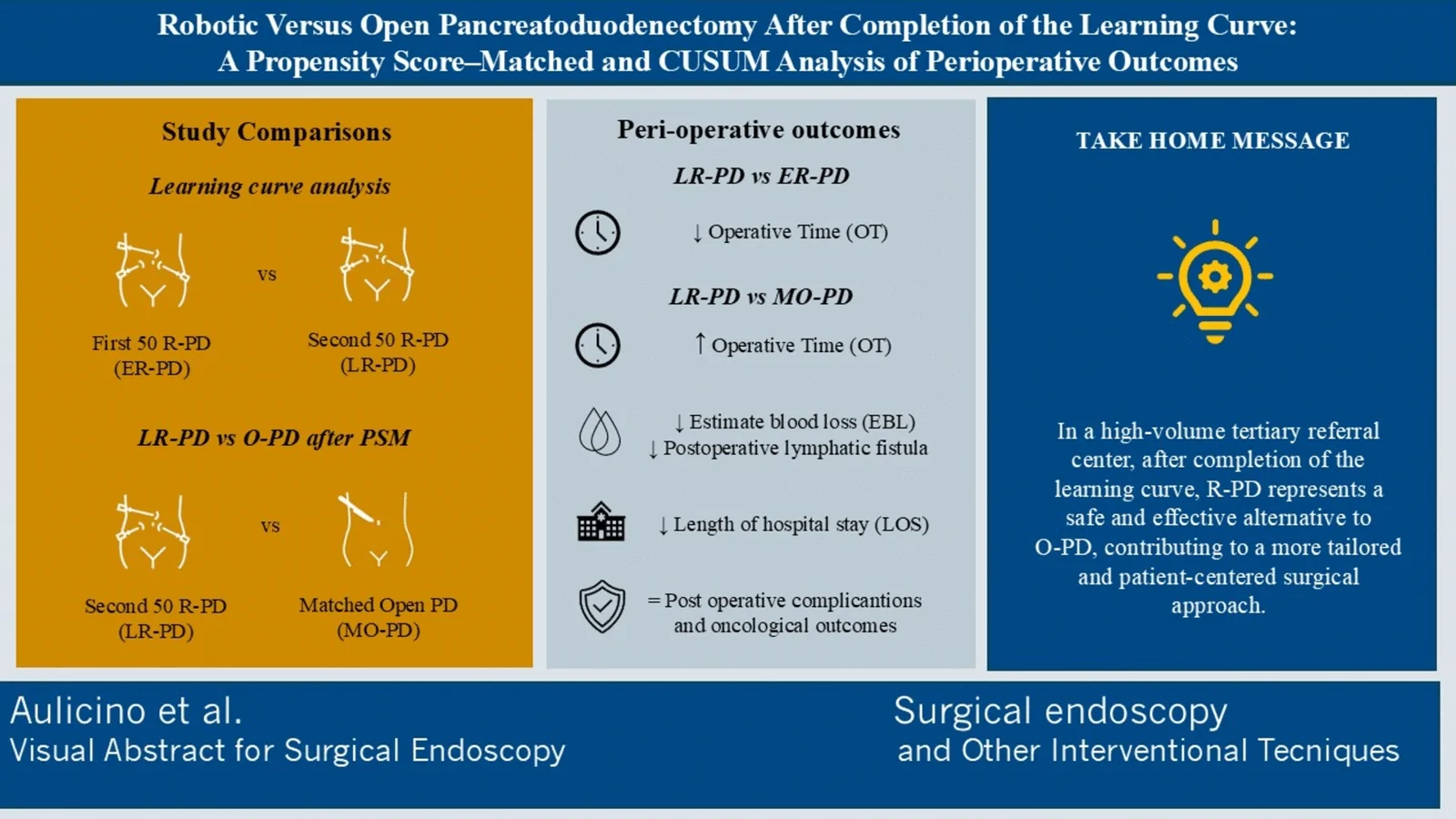

**Supplementary Information:**

The online version contains supplementary material available at https://doi.org/10.1007/s00464-026-13143-3.

Pancreatoduodenectomy (PD) is one of the most complex procedures in abdominal surgery and represents the standard surgical treatment for malignant and selected benign diseases of the pancreatic head and periampullary region [1–4]. Despite substantial improvements in surgical technique, perioperative management, and the centralization of pancreatic surgery, PD remains associated with considerable morbidity, even in high-volume centers [5, 6].

In recent years, minimally invasive approaches have been increasingly adopted in pancreatic surgery with the aim of reducing surgical trauma while preserving oncological adequacy [7–9]. In this context, robotic pancreatoduodenectomy (R-PD) has gained growing attention as a potential alternative to conventional open pancreatoduodenectomy (O-PD) [10, 11]. The robotic platform offers several technical advantages, including three-dimensional high-definition visualization, tremor filtration, and articulated instruments, which may facilitate more precise dissection and the execution of complex reconstructive steps [12, 13].

Despite these potential benefits, the effectiveness of R-PD remains debated. Its adoption is still limited by the technical complexity of the procedure, longer operative times, and the substantial learning curve required to achieve stable perioperative outcomes [14–17]. Moreover, the currently available literature reports heterogeneous results compared with other approaches, likely reflecting differences in patient selection, institutional experience, and the inclusion of cases performed during the early phases of robotic program implementation [14, 15].

In this setting, comparative studies controlling for baseline differences between surgical approaches are essential to better define the role of R-PD in clinical practice. Propensity score–matched (PSM) analyses may help reduce selection bias and provide a more balanced comparison between robotic and open techniques.

Therefore, the aim of the present study was to compare perioperative outcomes between R-PD and O-PD in a high-volume tertiary referral center using a propensity score–matched cohort. To minimize the impact of the learning curve, the analysis was limited to cases performed by a single surgeon after completion of the initial robotic experience. In addition, a comparison of perioperative outcomes between the early and late phases of the robotic surgical experience was performed, together with a CUSUM analysis of the outcomes that differed between the early and late phases of the learning curve.

## Methods

This single-center propensity score–matched retrospective cohort study analyzed data from a prospectively maintained institutional database of patients undergoing R-PD or O-PD between January 2020 and December 2025 at the Pancreatic Surgery Unit of the Fondazione Policlinico Universitario A. Gemelli IRCCS of Rome.

Inclusion criteria were adult patients (≥ 18 years) diagnosed with pancreatic or periampullary diseases, including pancreatic ductal adenocarcinoma (PDAC), periampullary neoplasms (tumors of the ampulla of Vater or distal bile duct), cystic pancreatic neoplasms requiring surgical resection, pancreatic neuroendocrine tumors, and other benign or malignant conditions requiring pancreatic head resection.

All cases were evaluated by a multidisciplinary team (MDT) to determine surgical indication and resectability. All patients were thoroughly informed about the potential risks and benefits of the procedure, and written informed consent for surgery was obtained.

The following baseline characteristics were collected: age, sex, body mass index (BMI), American Society of Anesthesiologists (ASA) score [18], Charlson Comorbidity Index (CCI) [19], insulin-dependent diabetes, histological diagnosis, tumor size, tumor resectability according to National Comprehensive Cancer Network (NCCN) criteria [20] (resectable or borderline resectable), neoadjuvant treatment (NAT), vascular resection, pancreatic texture (soft or hard), main pancreatic duct diameter, and Fistula Risk Score (FRS) [21].

Perioperative variables included operative time (OT), estimated blood loss (EBL), perioperative blood transfusion, length of hospital stay (LOS), reintervention, and readmission rates. Postoperative complications were graded according to the Clavien–Dindo (CD) classification [22]. Pancreas-specific complications, including postoperative pancreatic fistula (POPF) [23], delayed gastric emptying (DGE) [24], bile leak [25], and postpancreatectomy hemorrhage (PPH) [26], were defined according to the International Study Group of Pancreatic Surgery (ISGPS) criteria. The occurrence of postoperative lymphatic (chylous) fistula was also recorded.

Histopathological outcomes included the number of harvested lymph nodes and resection margin status (R status), defined according to the Royal College of Pathologists criteria [27]. Postoperative mortality was defined as death occurring within 30 days after surgery.

### Study outcomes

The primary endpoint of the study was the comparison between O-PD and R-PD in terms of postoperative outcomes, including major postoperative complications (CD grade ≥ III), pancreas-specific complications, reintervention rate, readmission rate, and length of hospital stay.

Secondary endpoints included intraoperative and pathological outcomes, specifically OT, EBL, perioperative blood transfusion, number of harvested lymph nodes, and resection margin status.

For this analysis, only the most recent 50 consecutive R-PD procedures (late group: LR-PD) were included, based on evidence suggesting that approximately 40–60 cases are required to overcome the learning curve and achieve stable perioperative outcomes [16, 28, 29]. This approach was adopted to reduce the potential impact of the learning curve and to ensure a more homogeneous level of surgical proficiency.

To minimize selection bias, outcomes were compared after PSM. Propensity scores were estimated using a logistic regression model, with surgical approach (O-PD vs LR-PD) as the dependent variable and baseline characteristics as covariates. Matching was performed using a 2:1 nearest-neighbor method without replacement. The matched groups (matched open group: MO-PD and LR-PD) were subsequently compared in terms of baseline characteristics as well as intraoperative, postoperative, and pathological outcomes.

Outcomes showing statistically significant differences between groups were subsequently included in multivariable regression analyses to assess the independent effect of surgical approach and to identify additional factors associated with each outcome.

Furthermore, an additional supplementary comparison was also performed between the overall O-PD population and the overall R-PD population. Finally, in order to further evaluate the impact of the learning curve, a subgroup analysis was performed comparing the entire cohort of patients undergoing O-PD with the first 50 R-PD procedures (early robotic group: ER-PD) and the subsequent 50 cases (late robotic group: LR-PD), assessing differences in baseline characteristics as well as perioperative and pathological outcomes among the groups. Subsequently, a CUSUM analysis was performed for the outcomes that showed differences between the ER-PD and LR-PD groups in order to identify the turning point of the robotic learning curve and to evaluate the progressive improvement in surgical performance and perioperative outcomes with increasing operative experience.

### Robotic pancreatic surgery program and surgical training

The robotic pancreatic surgery program at our institution was initiated in 2023 within an established high-volume pancreatic surgery unit performing more than 80 PD annually.

Implementation of R-PD followed a structured and progressive institutional pathway supported by prior experience in advanced minimally invasive pancreatic procedures and extensive training in hepatopancreatobiliary surgery. The surgeon performing all R-PDs included in the present study had previously completed dedicated robotic surgical training and had gained progressive experience in complex robotic abdominal surgery before initiating the R-PD program.

During the initial phase of the program, patient selection was deliberately restricted to technically favorable cases, including absence of major vascular involvement, lower BMI, and tumors without locally advanced features. With increasing experience, indications were progressively expanded to include more complex resections requiring vascular reconstruction, reflecting maturation of the robotic program and completion of the learning curve.

Open pancreatoduodenectomies included during the study period were performed by experienced pancreatic surgeons within the same high-volume institutional setting according to standardized perioperative management protocols shared across the unit.

### Statistical analysis

Continuous variables were reported as median (minimum–maximum) and compared using the Student’s *t*-test or the Mann–Whitney *U* test, as appropriate based on data distribution. Categorical variables were expressed as frequencies and percentages and compared using the *χ*^2^ test.

Propensity score matching was performed using a logistic regression model, with surgical technique (O-PD vs LR-PD) as the dependent variable. Covariates included baseline characteristics showing a potential imbalance between groups (*p* ≤ 0.100). Propensity scores were matched using a 2:1 nearest-neighbor matching method without replacement.

Multivariable analyses were performed including variables with a univariate *p*-value ≤ 0.100. Logistic regression models were used for binary outcomes, whereas linear regression models were applied for continuous outcomes.

Cumulative sum (CUSUM) analyses were performed for the outcomes that showed differences or trends toward significance between the ER-PD and LR-PD groups (*p* ≤ 0.100).CUSUM curves were constructed by sequentially calculating the cumulative sum of the difference between each individual outcome and the overall cohort mean (for continuous variables) or expected event rate (for binary outcomes). The inflection point of each curve was considered indicative of the transition from the initial learning phase to a more stable phase of surgical performance.

Statistical significance was defined as a two-sided *p*-value < 0.05, and confidence intervals (CI) were calculated at the 95% level. All statistical analyses were performed using IBM SPSS Statistics version 25.

## Results

### Comparison of outcomes between unmatched O-PD and R-PD cohorts

During the study period, 398 consecutive PDs were performed at the Pancreatic Surgery Unit of the Fondazione Policlinico Universitario Agostino Gemelli IRCCS, Rome, Italy. Of these, 298 patients (74.9%) underwent O-PD and 100 (25.1%) R-PD.

Baseline characteristics of O-PD and R-PD cohorts are summarized in Supplementary Material 1. No statistically significant differences were observed between groups regarding age, sex, BMI, ASA score, CCI, pancreatic texture, Wirsung duct diameter, or FRS. However, patients undergoing R-PD presented significantly smaller tumors (25 (6–32) vs 28 (17–41); *p* = 0.041), lower rates of borderline-resectable disease (9 (9%) vs 57 (19.1%); *p* = 0.018), lower rates of NAT (13 (13%) vs 77 (25.8%); *p* = 0.007), and significantly fewer vascular resections (6 (6%) vs 62 (20.8%); *p* < 0.001) compared with the O-PD cohort.

Intraoperative and postoperative outcomes of O-PD and R-PD cohorts are reported in Supplementary Material 2.

OT was significantly longer in the R-PD group compared with O-PD (640 (479–980) vs 389 (210–760) minutes; *p* < 0.001), whereas EBL was significantly lower following R-PD (200 (0–1500) vs 350 (0–2400) mL; *p* < 0.001). In addition, R-PD was associated with a significantly shorter LOS (13 (5–94) vs 15 (6–91) days; *p* = 0.022). No statistically significant differences were observed between groups regarding harvested lymph nodes, R1 resection rate, perioperative blood transfusion, major postoperative complications, CR-PPH, CR-POPF, bile leak, DGE, reinterventions, or readmission rates.

### Comparison of outcomes between unmatched O-PD cohort and early and late phases of R-PD

Baseline characteristics of patients undergoing O-PD, ER-PD and LR-PD are reported in Table 1.

**Table 1:** Baseline characteristics comparison between unmatched O-PD, ER-PD, and LR-PD cohorts

Variable	O-PD 298	ER-PD 50	LR-PD 50	P (O vs ER)	P (O vs LR)	P (ER vs LR)
Age, median (min–max)	68 (31–85)	67 (18–79)	66.5 (40–80)	0.458	0.114	0.909
Gender, *n* (%)				0.404	0.449	0.224
Male	172 (57.71)	32 (64)	26 (52)			
Female	126 (42.28)	18 (36)	24 (48)			
BMI, median (min–max)	24.87 (17–38)	24.12 (18–34)	23.72 (19–37)	0.586	0.387	0.547
ASA, *n* (%)				0.432	0.837	0.656
< 3	204 (68.45)	37 (74)	35 (70)			
≥ 3	94 (31.54)	13 (26)	15 (30)			
CCI, median (min–max)	5 (2–10)	4 (3–7)	4 (2–7)	0.295	0.214	0.709
Insulin-dependent diabetes, *n* (%)				0.748	0.362	0.662
Yes	77 (25.83)	14 (28)	16 (32)			
No	221 (74.16)	36 (72)	34 (68)			
Hystological type, *n* (%)						
PDAC of the pancreatic head	229 (76.84)	33 (66)	35 (70)	0.092	0.245	0.668
Ampully tumor	10 (3.35)	2 (4.25)	3 (6.38)	0.611	0.288	0.645
IPMN	19 (6.37)	5 (10.64)	4 (8.51)	0.215	0.585	0.725
Distal cholangiocarcinoma	10 (3.35)	2 (4)	2 (4)	0.808	0.534	1
Other	30 (10.06)	8 (17.02)	6 (12.76)	0.119	0.573	0.562
Tumor size, *n* (%)	28 (17–41)	23.5 (6–31)	26 (16–32)	**0.038**	**0.095**	0.117
Bordeline-resectable, *n* (%)				**0.023**	0.225	0.294
Yes	57 (19.12)	3 (6)	6 (12)			
No	241 (80.87)	47 (94)	44 (88)			
Neoadjuvant treatment, *n* (%)				**0.034**	**0.097**	0.766
Yes	77 (25.83)	6 (12)	7 (14)			
No	221 (74.16)	44 (88)	43 (86)			
Vascular resection, *n* (%)				**0.002**	**0.072**	0.092
Yes	62 (20.8)	1 (2)	5 (10)			
No	236 (79.19)	48 (98)	45 (90)			
Pancreatic texture, *n* (%)				0.953	0.829	0.826
Soft	106 (35.57)	18 (36)	17 (34)			
Hard	192 (64.42)	32 (64)	33 (66)			
Wirsung diameter, median (min–max)	4 (1–12)	4 (1–10)	3.5 (1–10)	0.864	0.923	0.833
Fistula risk score, median (min–max)	9 (4–14)	7 (3–13)	8 (3–14)	0.128	0.256	0.799

Statistical significance was set at *p* < 0.05): statistically significant values are given in bold

*BMI* body mass index, *ASA* American Society of Anesthesiologists Score, *CCI* Charlson Comorbidity Index, *PDAC* pancreatic ductal adenocarcinoma, *IPMN* Intraductal papillary mucinous neoplasm

Compared with the O-PD cohort, patients undergoing ER-PD showed significantly smaller tumor size (23.5 (6–31) vs 28 (17–41); *p* = 0.038), lower rates of borderline-resectable disease (3 (6%) vs 57 (19.2%); *p* = 0.023), lower rates of neoadjuvant treatment (6 (12%) vs 77 (25.83%); *p* = 0.034), and significantly fewer vascular resections (1 (2%) vs 62 (20.8%); *p* = 0.002). However, when comparing O-PD with LR-PD, these differences did not reach statistical significance. No significant differences were found among groups regarding age, sex, BMI, ASA score, CCI, Wirsung duct diameter, or FRS.

When comparing the two robotic subgroups, no statistically significant differences were observed in demographic, clinical, or tumor related characteristics. However, a non-significant trend toward larger tumor size (23.5 (6–31) vs 26 (16–32); *p* = 0.117), and an hight rate of vascular resections (1 (2%) vs 5 (10%); *p* = 0.092) was observed in the LR-PD group.

Conversion to open surgery occurred in 3 of 50 ER-PDs (6%) and 3 of 50 LR-PDs (6%) because of intraoperative technical difficulties and/or oncological concerns.

Intraoperative and postoperative outcomes of patients undergoing O-PD, ER-PD and LR-PD are summarized in Table 2.

**Table 2:** Intraoperative and postoperative outcomes of unmatched O-PD, ER-PD and R-PD cohorts

Variable	O-PD 298	ER-PD 50	LR-PD 50	P (O vs ER)	P (O vs LR)	P (ER vs LR)
OT, median (min–max)	389 (210–760)	705 (497–980)	582 (479–810)	** < 0.001**	** < 0.001**	** < 0.001**
EBL, median (min–max)	350 (0–2400)	200 (0–1100)	200 (0–1500)	**0.011**	**0.006**	0.397
Harvested nodes, median (min–max)	19 (6–72)	17 (9–38)	18 (11–43)	0.283	0.365	0.412
R1, *n* (%)	89 (29.86)	12 (24)	14 (28)	0.397	0.789	0.674
Peri-operative blood transfusion, *n* (%)				0.516	0.219	0.663
Yes	91 (30.53)	13 (26)	11 (22)			
No	207 (70.14)	37 (74)	39 (78)			
Major complications (CD grade ≥ III), *n* (%)	116 (38.92)	23 (46)	15 (30)	0.227	0.359	0.099
Grade IIIa	61 (20.46)	11 (22)	8 (16)	0.307	0.463	0.444
Grade IIIb	27 (9.06)	3 (6)	3 (6)	0.475	0.475	1.000
Grade IV	16 (5.36)	7 (14)	2 (4)	**0.023**	0.685	0.088
Grade V/30-day mortality	12 (4.03)	2 (4)	2 (4)	0.992	0.992	1.000
PPH grade B/C, *n* (%)	45 (15.10)	14 (28)	7 (14)	**0.024**	0.839	0.076
Grade B	28 (9.39)	9 (18)	4 (8)	0.067	0.751	0.137
Grade C	17 (5.70)	5 (10)	3 (6)	0.248	0.933	0.460
POPF grade B/C, *n* (%)	87 (29.19)	15 (30)	14 (28)	0.907	0.863	0.826
Grade B	75 (25.17)	13 (26)	11 (22)	0.900	0.630	0.640
Grade C	12 (4.03)	2 (4)	3 (6)	0.992	0.524	0.646
Post operative bile leak grade B/C, *n* (%)	24 (8.05)	6 (12)	4 (8)	0.357	0.989	0.504
Grade B	14 (4.70)	4 (8)	3 (6)	0.329	0.692	0.695
Grade C	10 (3.36)	2 (4)	1 (2)	0.817	0.612	0.557
DGE grade B/C, *n* (%)	62 (20.80)	14 (28)	9 (18)	0.254	0.648	0.234
Grade B	47 (15.77)	10 (20)	6 (12)	0.454	0.492	0.275
Grade C	15 (5.03)	4 (8)	3 (6)	0.392	0.775	0.695
Post operative lymphatic fistula, *n* (%)				0.492	**0.026**	0.140
Yes	47 (15.77)	6 (12)	2 (4)			
No	253 (84.23)	44 (88)	48 (96)			
Re-interventions, *n* (%)	116 (38.92)	23 (46)	17 (34)	0.344	0.507	0.220
Radiological	51 (19.46)	10 (20)	9 (18)	0.619	0.878	0.799
Endoscopic	21 (7.38)	3 (6)	2 (4)	0.511	0.422	0.646
Surgical reoperation	34 (11.41)	10 (20)	6 (12)	0.091	0.903	0.275
LOS, median (min–max)	15 (6–91)	14 (5–94)	12 (6–40)	0.492	**0.019**	0.154
Re-admission, *n* (%)	65 (21.81)	10 (20.0)	11 (22)	0.773	0.976	0.806

Statistical significance was set at *p* < 0.05): statistically significant values are given in bold

*OT* operative time, *EBL* estimate blood loss, *CD* Clavien Dindo, *PPH* postpancreatectomy haemorrhage, *POPF* postoperative pancreatic fistula, *DGE* delayed gastric emptying, *LOS* lenght of stay

OT was significantly longer in both ER-PD and LR-PD than in O-PD (705 (497–980) vs 389 (210–760) minutes; *p* < 0.001 and 582 (479–810) vs 389 (210–760) minutes; *p* < 0.001, respectively). Nevertheless, a significant reduction in OT was observed between the ER-PD and LR-PD groups (705 (497–980) vs 582 (479–810) minutes; *p* < 0.001).

EBL was significantly lower in both robotic groups than in O-PD (200 (0–1100) vs 350 (0–2400) mL; *p* = 0.011 for ER-PD and 200 (0–1500) vs 350 (0–2400) mL; *p* = 0.006 for LR-PD), whereas no statistically significant difference emerged between ER-PD and LR-PD (*p* = 0.397).

The ER-PD group showed significantly higher rates of CD grade IV complications (7 (14%) vs 16 (5.36%); *p* = 0.023) and clinically relevant PPH (14 (28%) vs 45 (15.1%); *p* = 0.024) than O-PD, while these differences were no longer evident in the LR-PD cohort.

Conversely, LR-PD was associated with a significantly lower rate of postoperative lymphatic fistula (1 (4%) vs 47 (15.77%); *p* = 0.026) and a shorter LOS (12 (6–40) vs 15 (6–91) days; *p* = 0.019) relative to O-PD.

Within the robotic cohort, a non-significant trend toward higher rates of major postoperative complications (CD ≥ III) was observed in the ER-PD compared with the LR-PD (23 (46% vs 15 (30%); *p* = 0.099), with more evident differences for CD grade IV complications (7 (14%) vs 2 (4%); *p* = 0.088) and CR-PPH (14 (28%) vs 7 (14%); *p* = 0.076).

No significant differences were observed among groups regarding harvested lymph nodes, R1 resection rate, perioperative blood transfusion, CR-POPF, bile leak, DGE, reinterventions, or readmission rates.

### CUSUM analysis of outcomes showing differences between early and late phases of the R-PD learning curve

#### Operative time

**Fig. 1:**
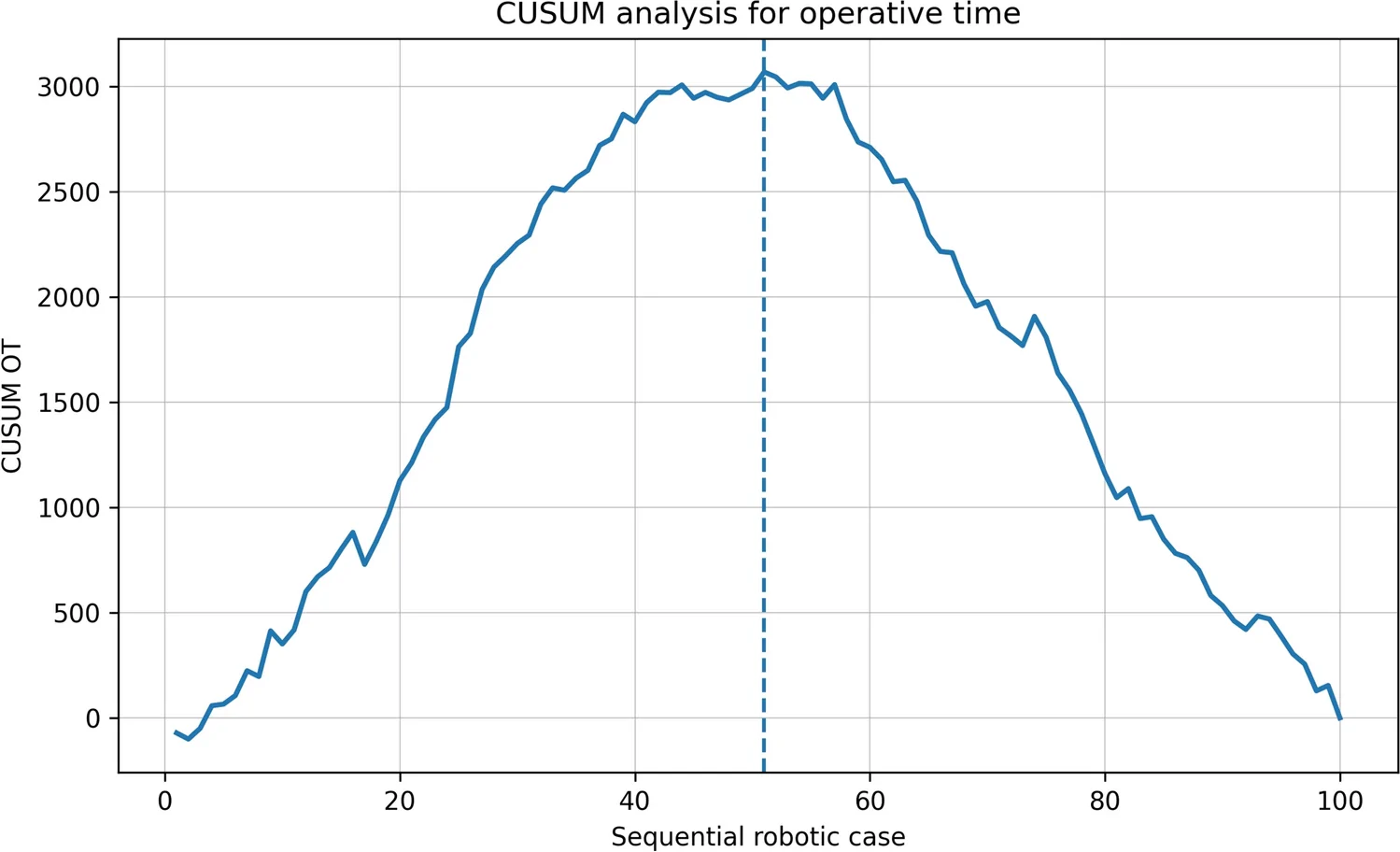
Flow chart of patient’s selection

CUSUM analysis of OT demonstrated a clear learning curve effect throughout the study period (Fig. 1). During the initial phase of the robotic experience, the curve showed a progressive upward trend, reflecting OT above the cohort mean and greater variability between consecutive procedures. This phase was followed by a plateau and subsequent downward slope, indicating progressive improvement in operative efficiency and increasing procedural standardization. The main inflection point of the CUSUM curve was identified at case 51, suggesting transition from the initial learning phase to a more reproducible phase of operative performance.

#### Major complications

**Fig. 2:**
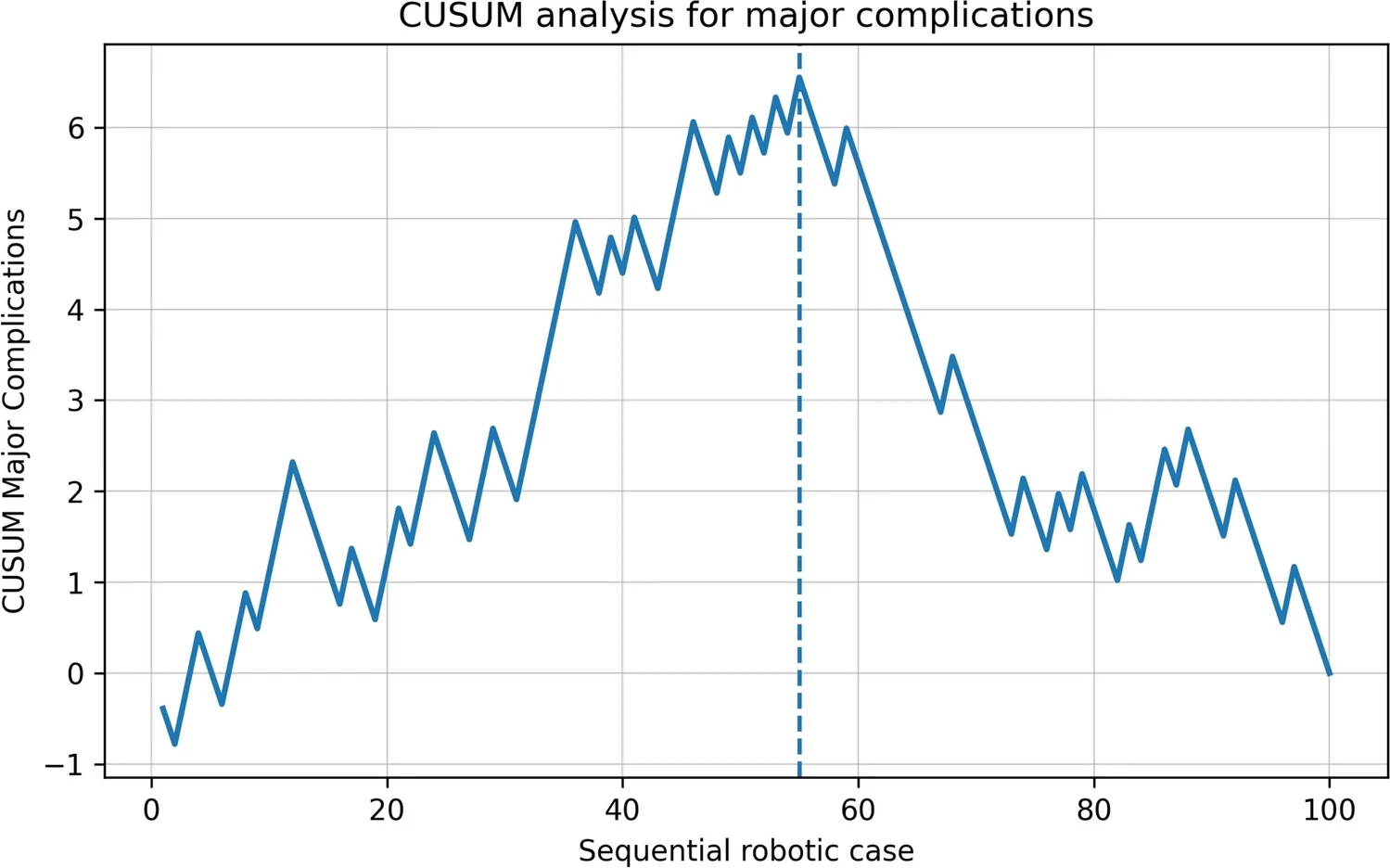
CUSUM analysis of operative time during the robotic pancreatoduodenectomy learning curve

CUSUM analysis for major postoperative complications (CD ≥ III) also demonstrated a learning curve effect during the study period (Fig. 2). The curve showed a progressive increase during the initial and intermediate phases of the robotic experience, indicating accumulation of major postoperative events above the expected cohort rate. After reaching the main inflection point at case 55, the curve progressively stabilized and subsequently declined, suggesting improvement in postoperative outcomes with increasing surgical experience.

#### Clinically relevant PPH

**Fig. 3:**
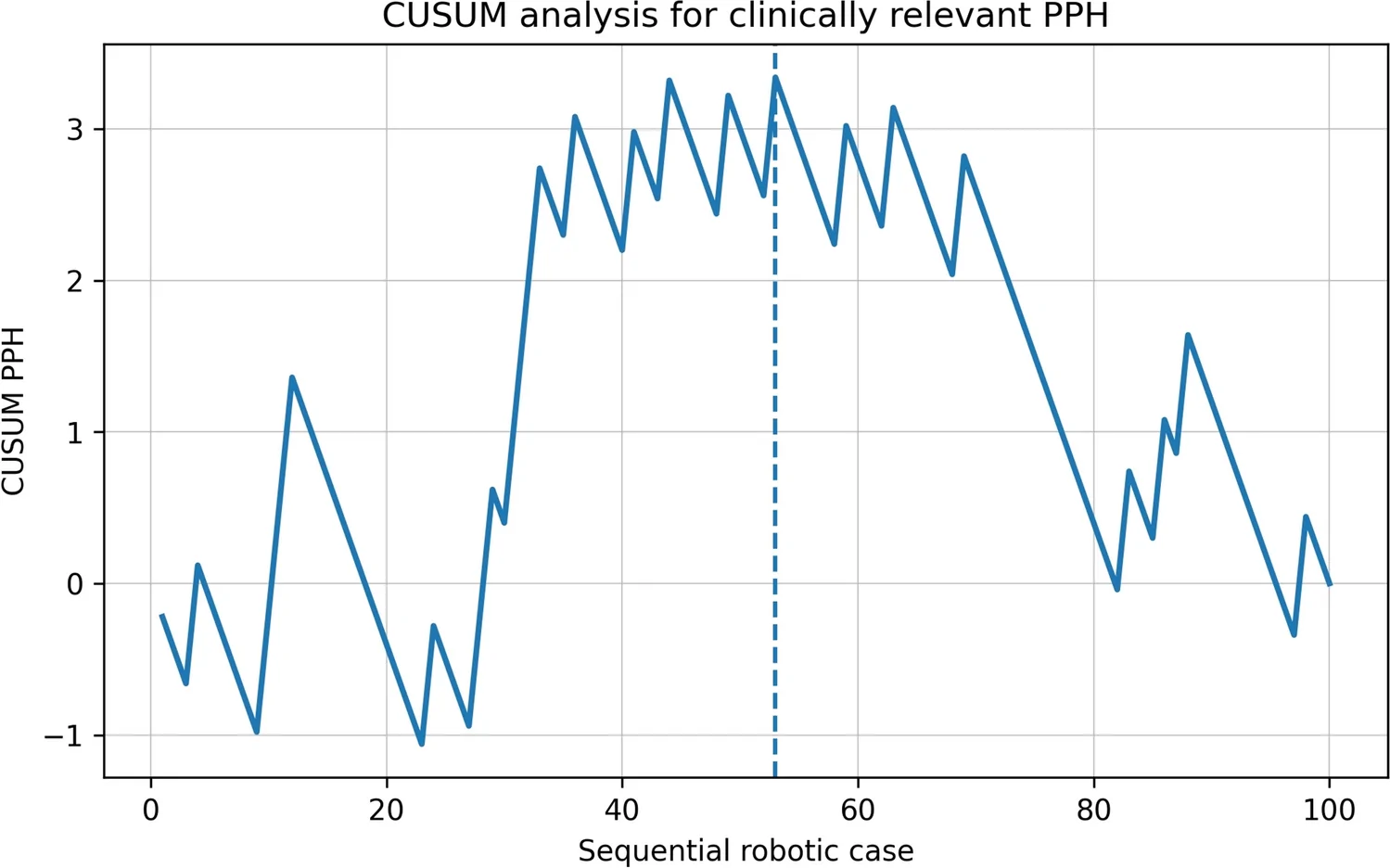
CUSUM analysis of major postoperative complications (Clavien–Dindo ≥ III) during the robotic pancreatoduodenectomy learning curve

CUSUM analysis for CR-PPH demonstrated a similar trend throughout the robotic experience (Fig. 3). During the initial phase, the curve showed a progressive upward slope, reflecting a higher incidence of clinically relevant hemorrhagic events. Following the main inflection point identified at case 53, the curve progressively stabilized and subsequently declined, suggesting improved bleeding control and increasing technical proficiency during the later phase of the robotic learning curve.

### Post-PSM comparison of perioperative outcomes between MO-PD and LR-PD

**Fig. 4:**
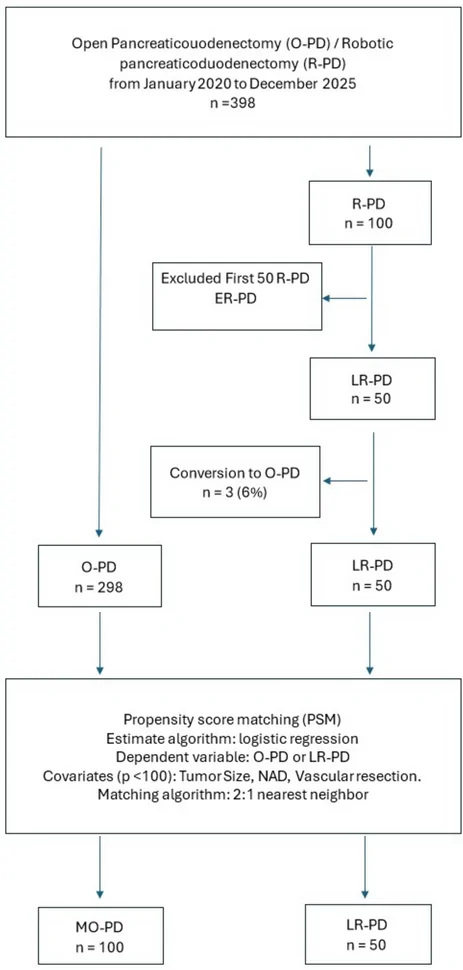
CUSUM analysis of clinically relevant postpancreatectomy hemorrhage (CR-PPH) during the robotic pancreatoduodenectomy learning curve

To minimize the impact of baseline imbalances between the O-PD and LR-PD cohorts, a PSM analysis was performed. As reported in Table 1, vascular resection (62 (20.8%) vs 5 (10%); *p* = 0.072), tumor size (28 (17–41) vs 26 (16–32); *p* = 0.095) and NAT (77 (25.83%) vs 7 (14%); *p* = 0.097) showed a trend toward imbalance. These variables were therefore included as covariates in the propensity score model (Fig. 4).

After PSM, 100 patients in the MO-PD group were compared with 50 patients in the LR-PD group. Post-matching analysis confirmed adequate balance between groups, with no statistically significant differences in baseline characteristics (Supplementary Material 3).

Intraoperative and postoperative outcomes are reported in Table 3. MO-PD was associated with a significantly shorter OT compared with LR-PD (362 (210–680) vs 582 (479–810) minutes; *p* < 0.001), whereas EBL was significantly lower in the LR-PD group (200 (0–1500) vs 300 (0–2100) mL; *p* = 0.007). No significant differences were observed in the number of harvested lymph nodes and R status. Rates of perioperative blood transfusion and CD ≥ III complications were comparable between groups. Similarly, no significant differences were found in terms of CR-POPF, PPH, bile leak, or DGE. A significantly lower incidence of postoperative lymphatic fistula was observed in the LR-PD group compared with the MO-PD group (2 (4%) and 15 (15%), respectively; *p* = 0.045).

**Table 3:** Intraoperative and postoperative outcomes of MO-PD vs LR-PD

Variable	MO-PD 100	LR-PD 50	*P*-value
OT, median (min–max)	362 (210–680)	582 (479–810)	** < 0.001**
EBL, median (min–max)	300 (0–2100)	200 (0–1500)	**0.012**
Harvested nodes, median (min–max)	20 (6–69)	18 (11–43)	0.348
R1, *n* (%)	28 (28)	15 (30)	0.798
Peri-operative blood transfusion, *n* (%)			0.685
Yes	25 (25)	11 (22)	
No	75 (75)	39 (78)	
Major complications (CD grade ≥ III), *n* (%)	36 (36)	16 (32)	0.809
Grade IIIa	19 (19)	9 (18)	0.882
Grade IIIb	8 (8)	3 (6)	0.657
Grade IV	4 (4)	2 (4)	1
Grade V/30-day mortality	5 (5)	2 (4)	0.784
PPH grade B/C, *n* (%)	13 (13)	7 (14)	0.865
Grade B	8 (8)	4 (8)	1
Grade C	5 (5)	3 (6)	0.797
POPF grade B/C, *n* (%)	28 (28)	14 (28)	1
Grade B	25 (25)	11 (22)	0.685
Grade C	4 (4)	3 (6)	0.584
Post Operative Bile leak grade B/C, *n* (%)	7 (7)	4 (8)	0.824
Grade B	4 (4)	3 (6)	0.584
Grade C	3 (3)	1 (2)	0.720
DGE grade B/C, *n* (%)	19 (19)	9 (18)	0.882
Grade B	15 (15)	6 (12)	0.617
Grade C	5 (5)	3 (6)	0.797
Post operative lymphatic fistula, *n* (%)			
Yes	15 (15)	2 (4)	**0.045**
No	85 (85)	48 (96)	
Re-interventions, *n* (%)	36 (36)	17 (34)	0.809
Radiological	18 (18)	9 (18)	1.000
Endoscopic	7 (7)	2 (4)	0.466
Surgical reoperation	11 (11)	6 (12)	0.855
LOS, median (min–max)	14 (6–91)	12 (6–40)	**0.038**
Re-admission, *n* (%)	20 (20)	11 (22)	0.676

Statistical significance was set at *p* < 0.05): statistically significant values are given in bold

*OT* operative time, *EBL* estimate blood loss, *CD* Clavien Dindo, *PPH* postpancreatectomy haemorrhage, *POPF* postoperative pancreatic fistula, *DGE* delayed gastric emptying, *LOS* lenght of stay

Reintervention rates (radiological, endoscopic, or surgical) and hospital readmission were similar between groups. LOS was significantly shorter in the LR-PD group compared with the MO-PD group (12 (6–36) vs 14 (6–91) days; *p* = 0.038).

### Multivariable analysis of predictors of perioperative outcomes in the matched population

The results of the univariate and multivariable analyses for OT and EBL are reported in Table 4.

**Table 4:** Univariate and multivariable analyses of factors associated with OT and EBL in the matched population

	All patients *N* 150	Prognostic factors analysis for OT (*multivariable linear regression*)				Prognostic factors analysis for EBL (*multivariable linear regression*)			
		Univariate Analysis		Multivariate Analysis		Univariate Analysis		Multivariate Analysis	
Variable	*N* (%)/median (min–max)	*B* [95% CI]	*P* value	*B* [95% CI]	*P* value	*B* [95% CI]	*P* value	*B* [95% CI]	*P* value
Age	67 (31–83)	1.14 [0.96–3.24]	0.286			0.96 [− 1.23–3.89]	0.386		
Gender									
Male	82 (54.66)	17.20 [1.45–33.95]	**0.044**	8.95 [− 7.78–25.65]	0.270	72.18 [9.24–132.58]	**0.027**	20.46 [− 36.20–89.67]	0.477
Female	68 (45.33)								
BMI	23.93 (17–37)	1.86 [− 1.92–5.64]	0.333			2.18 [− 3.88–8.24]	0.481		
ASA score									
I-II	104 (69.33)								
III	46 (30.66)	16.80 [1.35–32.25]	**0.034**	14.70 [2.36–27.64]	**0.026**	37.66 [− 18.32–93.50]	0.187		
Charlson Comorbidity Index	4.5 (2–9)	1.62 [− 1.74–7.94]	0.336			8.40 [− 7.20–24.00]	0.290		
Insulin-dependent diabetes									
Yes	42 (28)	7.26 [− 5.19–15.54]	0.288			53.20 [− 17.60–124.00]	0.140		
No	108 (72)								
Hystological Type									
PDAC of the pancreatic head*	109 (72.66)	*	*			*	*		
Ampully tumor	7 (4.66)	− 13.80 [− 41.62–6.26]	0.214			− 16.54 [− 59.83–13.23]	0.305		
IPMN	13 (8.66)	− 7.18 [− 22.44–13.90]	0.392			− 29.17 [− 70.86–16.91]	0.207		
Distal cholangiocarcinoma	6 (4)	2.25 [− 29.82–27.97]	0.561			− 10.12 [− 47.62–16.29]	0.482		
Other	15 (10)	5.40 [− 26.83–34.86]	0.681			− 6.807 [− 30.40–12.12]	0.601		
Surgical approach									
Robotic	50 (30)	105.65 [93.39–118.27]	** < 0.001**	99.75 [84.90–112.50]	** < 0.001**	−164.47 [−231.2–95.6]	** < 0.001**	− 116.80 [− 326.1– − 76.2]	**0.026**
Open	100 (70)								
Tumor size	26 (15–35)	1.18 [− 1.13–3.46]	0.307			3.15 [0.62–5.68]	**0.015**	1.02 [− 1.29–3.34]	0.386
Neoadjuvant treatment									
Yes	24 (16)	32.14 [9.73–58.59]	**0.012**	37.26 [5.33–69.17]	**0.022**	117.434 [22.82–209.02]	**0.016**	89.70 [62.23–189.63]	**0.041**
No	126 (84)								
Vascular resection									
Yes	16 (10.66)	35.10 [21.94–63.83]	**0.003**	61.86 [41.27–82.44]	** < 0.001**	352.63 [267.82–437.44]	** < 0.001**	315.05 [216.48–414.67]	** < 0.001**
No	134 (89.33)								
Harvested nodes	19.5 (6–69)	0.86 [− 0.88–2.61]	0.330			0.78 [− 1.90–3.46]	0.567		
Pancreatic texture									
Soft	53 (35.33)	10.46 [− 2.72–22.97]	0.121			134.92 [37.89–226.63]	**0.006**	144.23 [86.62–287.82]	**0.009**
Hard	97 (64.66)								
Wirsung diameter	4 (1–12)	1.05 [− 1.30–6.43]	0.380			− 0.58 [− 13.40–4.84]	0.349		
Fistula Risk Score	9 (3–14)	2.61 [− 0.76–11.93]	0.181			6.50 [− 5.83–18.84]	0.298		

Statistical significance was set at *p* < 0.05): statistically significant values are given in bold

*OT* operative time, *EBL* estimated blood loss, *BMI* body mass index, *ASA* American Society of Anesthesiologists score, *PDAC* pancreatic ductal adenocarcinoma, *IPMN* intraductal papillary mucinous neoplasm

^*^Reference category

#### Operative time

On univariate analysis, male sex (*B* = 17.20 [1.45–33.95]; *p* = 0.044), ASA score III–IV (*B* = 16.80 [1.35–32.25]; *p* = 0.034), LR-PD (*B* = 105.65 [93.39–118.27]; *p* = < 0.001), NAT (*B* = 32.14 [9.73–58.59]; *p* = 0.012), and vascular resection (*B* = 35.10 [21.94–63.83]; *p* = 0.003) were associated with longer OT.

After adjustment in the multivariable model, ASA score III–IV (*B* = 14.70 [2.36–27.64]; *p* = 0.026), LR-PD (*B* = 99.75 [84.90–112.50]; *p* < 0.001), NAT (*B* = 37.26 [5.33–69.17]; *p* = 0.022), and vascular resection (*B* = 61.86 [41.27–82.44]; *p* < 0.001) remained independently associated with longer OT.

#### Estimated blood loss

At univariate analysis, male sex (*B* = 72.18 [9.24–132.58]; *p* = 0.027), tumor size (*B* = 3.15 [0.62–5.68]; *p* = 0.015), NAT (*B* = 117.434 [22.82–209.02]; *p* = 0.016, vascular resection (*B* = 352.63 [267.82–437.44]; *p* < 0.001, and soft pancreatic texture (*B* = 134.92 [37.89–226.63]; *p* = 0.006) were associated with higher EBL, whereas LR-PD (*B* = −164.47 [−231.2–95.6]; *p* < 0.001) was associated with lower EBL.

In the multivariable model, the robotic approach remained independently associated with lower EBL compared with the open approach (*B* = − 116.80 [− 326.1 to − 76.2]; *p* = 0.026), whereas NAT (*B* = 89.70 [62.23–189.63]; *p* = 0.041), vascular resection (*B* = 315.05 [216.48–414.67]; *p* < 0.001), and soft pancreatic texture (*B* = 144.23 [86.62–287.82]; *p* = 0.009) were independently associated with increased EBL.

### Multivariable analysis of predictors of length of hospital stay

Factors associated with prolonged LOS are reported in Table 5.

**Table 5:** Univariate and multivariable analyses of factors associated with LOS in the matched population

	All patients *N* 150	Prognostic factors analysis for length of hospitalization (*multivariable linear regression*)			
		Univariate analysis		Multivariate analysis	
Variable	*N* (%)/median (min–max)	*B* [95% CI]	*P* value	*B* [95% CI]	*P* value
Age	67 (31–83)	0.09 [−0.03–0.13]	0.401		
Gender		0.79 [−0.31–2.47]	0.191		
Male	82 (54.66)				
Female	68 (45.33)				
BMI	23.93 (17–37)	0.16 [−0.09–0.31]	0.387		
ASA score					
I-II	104 (69.33)				
III	46 (30.66)	1.59 [0.21–2.98]	**0.031**	1.31 [0.19–2.27]	**0.013**
Charlson Comorbidity Index	4.5 (2–9)	0.19 [−0.21–0.59]	0.247		
Insulin-dependent diabetes					
Yes	42 (28)	1.46 [−0.81–2.92]	0.156		
No	108 (72)				
Hystological type					
PDAC of the pancreatic head *	109 (72.66)	*	*		
Ampully tumor	7 (4.66)	− 0.61 [–1.93–1.68]	0.289		
IPMN	13 (8.66)	− 0.76 [–1.25–1.12]	0.192		
Distal cholangiocarcinoma	6 (4)	0.37 [−0.19–1.24]	0.521		
Other	15 (10)	0.29 [−0.29–1.64]	0.512		
Surgical approach					
Robotic	50 (30)	− 1.82 [–2.85–−0.63]	** < 0.001**	− 1.48 [−2.38 – −0.39]	**0.041**
Open	100 (70)				
Tumor size	26 (15–35)	0.03 [−0.01–0.12]	0.268		
Neoadjuvant treatment					
Yes	24 (16)	1.19 [−0.13–2.47]	**0.081**	0.91 [−0.67–1.77]	0.318
No	126 (84)				
Vascular resection					
Yes	16 (10.66)	2.29 [0.69–3.93]	**0.021**	1.58 [−0.54–2.35]	0.231
No	134 (89.33)				
Harvested nodes	19.5 (6–69)	0.05 [−0.04–0.17]	0.587		
Pancreatic texture					
Soft	53 (35.33)	0.73 [−0.39–2.36]	0.219		
Hard	97 (64.66)				
Wirsung diameter	4 (1–12)	0.04 [−0.19–0.41]	0.607		
Fistula risk score	9 (3–14)	0.25 [−0.25–0.78]	0.391		
OT	428 (210–810)	0.008 [0.004–0.009]	** < 0.001**	0.005 [0.002–0.008]	**0.017**
EBL	250 (0–2100)	0.002 [0.001–0.005]	**0.001**	0.0014 [0.0003–0.0024]	**0.010**
Peri-operative blood transfusion					
Yes	36 (24)	2.04 [1.12–3.01]	**0.024**	1.29 [−0.98–2.31]	0.142
No	114 (76)				
Major complication (CD ≥ 3)					
Yes	52 (34.66)	5.22 [2.11–6.96]	** < 0.001**	4.03 [1.08–6.92]	** < 0.001**
No	98 (65.33)				

Statistical significance was set at *p* < 0.05): statistically significant values are given in bold

*LOS* lenght of stay, *BMI* body mass index, *ASA* American Society of Anesthesiologists score, *PDAC* pancreatic ductal adenocarcinoma, *IPMN* intraductal papillary mucinous neoplasm, *OT* operative time, EBL estimate blood loss, *CD* Clavien Dindo

^*^Reference category

On univariate analysis, ASA score III–IV (*B* = 1.59 [0.21–2.98]; *p* = 0.031), vascular resection (*B* = 2.29 [0.69–3.93]; *p* = 0.021), OT (*B* = 0.008 [0.004–0.009]; *p* < 0.001), EBL (*B* = 0.002 [0.001–0.005]; *p* = 0.001), perioperative blood transfusion (*B* = 2.04 [1.12–3.01]; *p* = 0.024), and CD ≥ III complications (*B* = 5.22 [2.11–6.96]; *p* < 0.001) were associated with prolonged LOS. Conversely, the robotic surgical approach was associated with a shorter LOS (*B* = −1.82 [–2.85 to −0.63]; *p* < 0.001).

Following adjustment in the multivariable model, ASA score III–IV (*B* = 1.31 [0.19–2.27]; *p* = 0.013), OT (*B* = 0.005 [0.002–0.008]; *p* = 0.017), EBL (*B* = 0.0014 [0.0003–0.0024]; *p* = 0.010, and CD ≥ III complications (*B* = 4.03 [1.08–6.92], *p* < 0.001) were confirmed as independent predictors of prolonged LOS. In contrast, LR-PD remained independently associated with reduced LOS (*B* = −1.48 [−2.38 to −0.39]; *p* = 0.041).

### Multivariable analysis of predictors of chylous fistula

The variables associated with the development of postoperative chylous fistula are reported in Table 6.

**Table 6:** Univariate and multivariable analyses of factors associated with lymphatic fistula in the matched population

Variable	Chyle Leak (+) *N* 17 pts	Chyle Leak (−) *N* 133 pts	Prognostic factors analysis for postoperative chile leak (*multivariable logistic regression*)				
			Univariate Analysis		Multivariate Analysis		
	*N* (%)/Median (min–max)	*N* (%)/median (min–max)	OR [95% CI]	*P* value	OR [95% CI]		*P* value
Age	69 (45–81)	66 (31–83)	1.04 [0.72–1.11]	0.164			
Gender							
Male	11 (64.70)	71 (53.38)	1.89 [0.68–4.94]	0.221			
Femal	6 (35.29)	59 (46.61)					
BMI	24.92 (19–32)	24.22 (17–37)	1.05 [0.79–1.21]	0.481			
ASA score							
I-II	9 (53)	95 (71.42)					
III	8 (47)	38 (28.57)	2.21 [0.82–5.98]	**0.091**	1.57 [0.48–4.38]		0.368
Charlson Comorbidity Index	5 (2–9)	4 (2–8)	1.13 [0.86–1.33]	0.216			
Insulin-dependent diabetes							
Yes	6 (35)	36 (27.06)	1.55 [0.53–4.53]	0.409			
No	11 (65)	97 (72.93)					
Hystological type							
PDAC of the Pancreatic head	12 (70.6)	97 (72.93)	*	*			
Ampully tumor	1 (5.9)	6 (4.51)	1.47 [0.16–13.2]	0.716			
IPMN	1 (5.9)	12 (9.02)	0.67 [0.08–5.2]	0.793			
Distal cholangiocarcinoma	1 (5.9)	5 (3.75)	1.92 [0.17–17.9]	0.526			
Other	2 (11.8)	13 (9.7)	1.29 [0.23–7.1]	0.772			
Surgical approach							
Robotic	2 (11.8)	48 (36.09)	0.22 [0.08–0.97]	**0.034**	0.30 [0.09–0.99]		**0.047**
Open	15 (88.2)	85 (63.90)					
Tumor size	27 (18–35)	26 (15–34)	1.02 [0.92–1.07]	0.152			
Neoadjuvant Treatment							
Yes	6 (35.3)	18 (13.53)	3.32 [1.12–10.87]	**0.029**	3.02 [1.04–9.05]		**0.049**
No	11 (64.7)	115 (86.46)					
Vascular resection							
Yes	5 (29.4)	11 (8.27)	5.24 [1.38–18.80]	**0.013**	2.19 [0.74–9.13]		0.264
No	12 (70.6)	122 (91.73)					
Harvested nodes	22 (12–69)	18 (6–52)	1.07 [1.02–1.18]	**0.029**	1.03 [0.64–1.11]		0.307
Pancreatic texture							
Soft	5 (29.4)	48 (36.09)	1.52 [0.65–3.94]	0.534			
Hard	12 (70.6)	85 (63.90)					
Wirsung diameter	4 (2–11)	4 (1–12)	0.86 [0.63–1.21]	0.837			
Fistula risk score	9 (3–9)	8.5 (3–14)	1.08 [0.87–1.21]	0.446			

Statistical significance was set at *p* < 0.05): statistically significant values are given in bold

*BMI* body mass index, *ASA* American Society of Anesthesiologists score, *PDAC* pancreatic ductal adenocarcinoma, *IPMN* intraductal papillary mucinous neoplasm, *CD* Clavien Dindo

^*^Reference category

Univariate analysis identified NAT (OR = 3.32 [1.12–10.87]; *p* = 0.029), vascular resection (OR = 5.24 [1.38–18.80]; *p* = 0.013), and increasing number of harvested lymph nodes (OR = 1.07 [1.02–1.18]; *p* = 0.029) as related to a higher rate of chylous fistula, whereas the robotic surgical approach was associated with a significantly lower risk of this complication (OR = 0.22 [0.08–0.97]; *p* = 0.034).

When these variables were entered into the multivariable model, only robotic surgical approach (OR = 0.30 [0.09–0.99]; *p* = 0.047) and NAT (OR = 3.02 [1.04–9.05]; *p* = 0.049) remained independently associated with the occurrence of chylous fistula, with the robotic approach acting as a protective factor.

## Discussion

The present study provides a comparative analysis of R-PD versus MO-PD performed in a high-volume tertiary referral center, with the additional strength of controlling for selection bias through propensity score matching and minimizing the influence of the learning curve. According to our findings, LR-PD was associated with significantly lower EBL and shorter LOS, at the expense of longer OT, while no significant differences were observed in terms of major postoperative complications, pancreas-specific complications, or oncological outcomes.

These results are in line with the current body of evidence comparing minimally invasive and open approaches for PD [30, 31]. One of the most consistent findings in the literature is the trade-off between OT and perioperative benefits. In the present study, LR-PD was associated with a significantly longer OT compared with the open approach, which remained an independent predictor at multivariable analysis. This evidence has been consistently reported across both retrospective series and meta-analyses, with robotic procedures requiring on average 60–90 additional minutes [30, 31]. However, despite longer OT, robotic surgery appears to reduced LOS and EBL, likely due to improved visualization, enhanced dexterity, more precise dissection, and reduced surgical trauma.

In this context, the analysis of the learning curve within the robotic cohort provides additional insight into the evolution of operative performance over time. When comparing the early and late phases of the robotic experience, OT was significantly reduced in the LR-PD, while other intraoperative and postoperative outcomes remained comparable between groups. This finding was further supported by the CUSUM analysis for OT, which identified a clear inflection point at case 51, followed by a progressive downward trend of the curve, suggesting increasing operative efficiency and procedural standardization with growing surgical experience. A non-significant trend toward lower rates of major postoperative complications, including CD grade IV complications and clinically relevant PPH, was also observed in the late phase. Although these differences did not reach statistical significance, the corresponding CUSUM analyses demonstrated a progressive stabilization and subsequent decline of both major complications and clinically relevant PPH after cases 55 and 53, respectively. These results support the hypothesis that increasing robotic experience may contribute to improved perioperative management, bleeding control, and reduction of severe postoperative morbidity over time. Notably, these findings emerged despite a trend toward increased case complexity, characterized by larger tumor size and a higher rate of vascular resections, suggesting progressive expansion of surgical indications with increasing experience. These results are consistent with previous reports demonstrating improvement and stabilization of perioperative outcomes after completion of the learning curve for R-PD [16, 28, 29].

Nevertheless, the persistence of longer OT in the robotic group compared with the open approach, even after completion of the learning curve, deserves specific consideration. R-PD inherently includes additional procedural steps, such as docking and instrument exchange, which are not required in open surgery, while the fully intracorporeal reconstructive phase remains technically demanding and time-consuming even in experienced hands. Moreover, the later phase of the robotic experience was characterized by a progressive expansion of surgical indications toward more complex cases, which may have contributed to maintaining longer OT despite increasing surgical proficiency. Similar results have been reported in other high-volume series [30, 31], suggesting that OT may remain longer than in open surgery even after stabilization of perioperative outcomes and completion of the learning curve, without negatively affecting postoperative morbidity or oncological adequacy.

Two other important results of the present study are the significantly lower EBL and shorter LOS observed in the LR-PD group, both of which remained independently associated with the robotic approach after adjustment for potential confounding factors. This finding are strongly supported by the literature [30, 31]. In particular, a large meta-analysis including more than 50,000 patients reported significantly lower EBL and shorter LOS in the robotic group, while rates of major complications and oncological outcomes remained comparable between approaches [31]. From a clinical perspective, reduced EBL may translate into lower transfusion requirements and potentially improved postoperative recovery, although no significant differences in transfusion rates were observed in our cohort. Similarly, the shorter LOS associated with R-PD may reflect the minimally invasive nature of the procedure, resulting in reduced surgical trauma, faster recovery of gastrointestinal function, and earlier postoperative mobilization.

In contrast, no significant differences were observed between the two approaches in terms of major postoperative complications, including CR-POPF, DGE, PPH, and bile leak. These findings are consistent with both randomized and non-randomized studies [32, 33], which have demonstrated comparable perioperative safety between robotic and open pancreatoduodenectomy. Importantly, the absence of increased complication rates in the robotic group supports the safety of this approach when performed in experienced centers.

From an oncological standpoint, the two approaches yielded similar results in terms of lymph node harvest and R status. These findings are in line with previous studies demonstrating equivalent oncological adequacy between R-PD and O-PD [30, 34, 35]. The preservation of oncological outcomes is a critical prerequisite for the adoption of minimally invasive techniques in pancreatic surgery, and the present study further supports the oncological safety of R-PD in a high-volume setting.

An interesting finding of the present study concerns postoperative lymphatic/chylous fistula. In our analysis, the LR-PD was associated with a significantly lower incidence of lymphatic fistula compared with the open technique, and this protective effect was confirmed at multivariable analysis. Although data on this specific complication are relatively limited in the literature, this finding may be explained by the technical advantages of the robotic platform. Enhanced three-dimensional visualization and improved instrument articulation may allow for more precise identification and sealing of lymphatic vessels during lymphadenectomy and dissection along major vascular structures. In addition, the stability of the robotic platform may contribute to more meticulous tissue handling, potentially reducing lymphatic leakage.

These findings are in line with emerging evidence suggesting that minimally invasive approaches, particularly robotic surgery, may reduce certain procedure-specific complications through improved surgical precision. However, given the relatively low incidence of chylous fistula and the heterogeneity in its definition and reporting across studies, further investigations are warranted to confirm this potential benefit and to better elucidate the underlying mechanisms.

A key strength of the present study is the deliberate exclusion of the initial learning curve for R-PD from the comparative PSM comparison with the open approach. It is well established that outcomes of minimally invasive pancreatic surgery are highly dependent on surgeon experience and institutional volume [29, 32, 33, 36]. By including only cases performed after completion of the learning curve, the present analysis provides a more accurate representation of the “steady-state” performance of the robotic approach. This methodological choice likely reduces heterogeneity and enhances the validity of the comparison with open surgery.

In addition, the use of PSM represents another important strength, as it allows for adjustment of baseline differences between groups and reduces selection bias inherent to retrospective studies. Although randomized controlled trials remain the gold standard, they are still limited in this field, and high-quality observational studies with robust statistical adjustment continue to play a crucial role in informing clinical practice.

Despite these strengths, several limitations of the present study should be acknowledged. First, the retrospective design inherently exposes the analysis to residual confounding, even after PSM. Second, this is a single-center study conducted in a high-volume tertiary referral center, which may limit the generalizability of the results to lower-volume institutions. In addition, all R.PD were performed by a single experienced surgeon, whereas open procedures were carried out by multiple surgeons, potentially introducing a surgeon-related bias. Third, long-term oncological outcomes, including disease-free and overall survival, were not evaluated and therefore no conclusions can be drawn regarding the long-term effectiveness of the robotic approach. Finally, although the learning curve effect was minimized, the relatively small sample size of the robotic cohort may limit the statistical power for detecting differences in less frequent outcomes.

Therefore, despite the relevant findings of the present study, further prospective and multicenter investigations are required to confirm these results and to better define the role of robotic pancreatoduodenectomy in contemporary pancreatic surgical practice.

## Conclusion

In a high-volume tertiary referral center and after completion of the learning curve, R-PD represents a valid alternative to O-PD, being associated with reduced EBL, shorter LOS, and a lower incidence of postoperative lymphatic fistula, albeit at the cost of longer OT. Importantly, no differences were observed in major postoperative complications or oncological outcomes, supporting the safety and feasibility of the robotic approach in experienced centers.

## Supplementary Information

Below is the link to the electronic supplementary material.

Supplementary file1 (DOCX 18 KB)

Supplementary file2 (DOCX 19 KB)

Supplementary file3 (DOCX 18 KB)
