# Supplementary material for: Robotic versus open pancreatoduodenectomy after completion of the learning curve: a propensity score–matched and CUSUM analyses of perioperative outcomes
Source: Surg Endosc. 2026 Jul 23;40(10):8670–86. doi: 10.1007/s00464-026-13143-3 (PMC13633149; doi:10.1007/s00464-026-13143-3)
Supplement: Supplementary file 1 [file 464_2026_13143_MOESM1_ESM.docx]

| **Supplementary Material 1:** Baseline characteristics comparison between unmatched O-PD and R-PD cohorts | | | |
| --- | --- | --- | --- |
| **Variable** | **O-PD**  **298** | **R-PD**  **100** | **P-Value** |
| **Age, median (Min-Max)** **Gender, n (%)** Male Female **BMI, median (Min-Max)** **ASA, n (%)** < 3 ≥ 3 | 68 (31-85)  172 (57.71)  126 (42.28)  24.87 (17-38)  204 (68.45)  94 (31.54) | 67 (18-80)  58 (58)  42 (42)  23.93 (18-37)  72 (72)  28 (28) | 0.667  0.960  0.475  0.506 |
| **CCI, median (Min-Max)**  **Insulin-dependent diabetes, n (%)**  Yes  No  **Hystological Type, n (%)**  PDAC of the Pancreatic Head  Ampully Tumor  IPMN  Distal cholangiocarcinoma  Other  **Tumor size, n (%)**  **Bordeline-resectable, n (%)**  Yes  No  **Neoadjuvant Treatment, n (%)**  Yes  No  **Vascular resection, n (%)**  Yes  No  **Pancreatic texture, n (%)**  Soft  Hard  **Wirsung diameter, median (Min-Max)**  **Fistula Risk Score, median (Min-Max)** | 5 (2-10)  77 (25.83)  221 (74.16)  229 (76.84)  10 (3.35)  19 (6.37)  10 (3.35)  30 (10.06)  28 (17-41)  57 (19.12)  241 (80.87)  77 (25.83)  221 (74.16)  62 (20.8)  236 (79.19)  106 (35.57)  192 (64.42)  4 (1-12)  9 (4-14) | 4 (2-7)  30 (30)  70 (70)  68 (68)  5 (5)  9 (9)  4 (4)  14 (14)  25 (6-32)  9 (9)  91 (91)  13 (13)  87 (87)  6 (6)  94 (90)  35 (35)  65 (65)  3.5 (1-10)  8 (3-14) | 0.214  0.416  0.078  0.454  0.374  0.762  0.277  0.041  0.018  0.007  <0.001  0.917  0.712  0.394 |

BMI Body Mass Index. ASA American Society of Anesthesiologists Score. CCI Charlson Comorbidity Index. PDAC Pancreatic Ductal Adenocarcinoma. IPMN Intraductal Papillary Mucinous Neoplasm. Statistical significance was set at *p* < 0.05): statistically significant values are underlined.
