# Supplementary material for: Robotic versus open pancreatoduodenectomy after completion of the learning curve: a propensity score–matched and CUSUM analyses of perioperative outcomes
Source: Surg Endosc. 2026 Jul 23;40(10):8670–86. doi: 10.1007/s00464-026-13143-3 (PMC13633149; doi:10.1007/s00464-026-13143-3)
Supplement: Supplementary file 2 [file 464_2026_13143_MOESM2_ESM.docx]

| **Supplementary Material 2:** Intraoperative and postoperative outcomes of unmatched O-PD vs R-PD cohorts | | | |
| --- | --- | --- | --- |
| **Variable** | **O-PD 298** | **R-PD 100** | **P-Value** |
| **OT, median (Min-Max)** | 389 (210-760) | 640 (479–980) | <0.001 |
| **EBL, median (Min-Max)** | 350 (0-2400) | 200 (0–1500) | <0.001 |
| **Harvested nodes, median (Min-Max)** | 19 (6-72) | 18 (9–43) | 0.639 |
| **R1, n (%)** | 89 (29.86) | 26 (26) | 0.460 |
| **Peri-operative blood transfusion, n (%)** |  |  | 0.212 |
| Yes | 91 (30.53) | 24 (24) |  |
| No | 207 (70.14) | 76 (76) |  |
| **Major complications (CD grade ≥III), n (%)** | 116 (38.92) | 38 (38) | 0.869 |
| Grade IIIa | 61 (20.46) | 19 (19) | 0.750 |
| Grade IIIb | 27 (9.06) | 6 (6) | 0.336 |
| Grade IV | 16 (5.36) | 9 (9) | 0.195 |
| Grade V/30-day mortality | 12 (4.03) | 4 (4) | 0.990 |
| **PPH grade B/C, n (%)** | 45 (15.10) | 21 (21) | 0.229 |
| Grade B | 28 (9.39) | 13 (13) | 0.304 |
| Grade C | 17 (5.70) | 8 (8) | 0.413 |
| **POPF grade B/C, n (%)** | 87 (29.19) | 29 (29) | 0.970 |
| Grade B | 75 (25.17) | 24 (24) | 0.815 |
| Grade C | 12 (4.03) | 5 (5) | 0.677 |
| **Post Operative Bile leak grade B/C, n (%)** | 24 (8.05) | 10 (10) | 0.546 |
| Grade B | 14 (4.70) | 7 (7) | 0.372 |
| Grade C | 10 (3.36) | 3 (3) | 0.862 |
| **DGE grade B/C, n (%)** | 62 (20.80) | 23 (23) | 0.643 |
| Grade B | 47 (15.77) | 16 (16) | 0.956 |
| Grade C | 15 (5.03) | 7 (7) | 0.456 |
| **Post Operative Lymphatic Fistula, n (%)** |  |  |  |
| Yes | 47 (15.77) | 8 (8) | 0.054 |
| No | 253 (84.23) | 92 (92) |  |
| **Re-interventions, n (%)** | 116 (38.92) | 40 (40) | 0.849 |
| Radiological | 51 (19.46) | 19 (19) | 0.668 |
| Endoscopic | 21 (7.38) | 5 (5) | 0.473 |
| Surgical reoperation | 34 (11.41) | 16 (16) | 0.230 |
| **LOS, median (Min-Max)** | 15 (6-91) | 13 (5–94) | 0.022 |
| **Re-admission, n (%)** | 65 (21.81) | 21 (21) | 0.864 |

OT Operative Time. EBL Estimate Blood Loss. CD Clavien Dindo. PPH Postpancreatectomy Hemorrhage. POPF Postoperative Pancreatic Fistula. DGE Delayed Gastric Emptying. LOS Lenght of Stay. Statistical significance was set at *p* < 0.05): statistically significant values are underlined.
