# Supplementary material for: Robotic versus open pancreatoduodenectomy after completion of the learning curve: a propensity score–matched and CUSUM analyses of perioperative outcomes
Source: Surg Endosc. 2026 Jul 23;40(10):8670–86. doi: 10.1007/s00464-026-13143-3 (PMC13633149; doi:10.1007/s00464-026-13143-3)
Supplement: Supplementary file 3 [file 464_2026_13143_MOESM3_ESM.docx]

| **Supplementary Material 3:** Post-matching baseline characteristics of patients undergoing MO-PD and LR-PD | | | |
| --- | --- | --- | --- |
| **Variable** | **MO-PD**  **100** | **LR-PD**  **50** | **P-Value** |
| **Age, median (Min-Max)** **Gender, n (%)** Male Female **BMI, median (Min-Max)** **ASA, n (%)** < 3 ≥ 3 | 67.5 (31-83)  56 (56)  44 (44)  24.03 (17-36)  69 (69)  31 (31) | 66.5 (42-80)  26 (52)  24 (40)  23.72 (19-37)  35 (70)  15 (30) | 0.368  0.642  0.693  0.900 |
| **CCI, median (Min-Max)**  **Insulin-dependent diabetes, n (%)**  Yes  No  **Hystological Type, n (%)**  PDAC of the Pancreatic Head  Ampully Tumor  IPMN  Distal cholangiocarcinoma  Other  **Tumor size, n (%)**  **Bordeline-resectable, n (%)**  Yes  No  **Neoadjuvant Treatment, n (%)**  Yes  No  **Vascular resection, n (%)**  Yes  No  **Pancreatic texture, n (%)**  Soft  Hard  **Wirsung diameter, median (Min-Max)**  **Fistula Risk Score, median (Min-Max)** | 4.5 (2-9)  26 (26)  74 (74)  74 (74)  4 (4)  9 (9)  4 (4)  9 (9)  26 (15-35)  14(14)  86 (86)  17 (17)  83 (83)  11 (11)  89 (89)  36 (36)  64 (64)  4 (1-12)  9 (4-13) | 4 (2-7)  16 (32)  34 (68)  35 (70)  3 (6.38)  4 (8.51)  2 (4)  6 (12.76)  26 (16-32)  6 (12)  44 (88)  7 (14)  43 (86)  5 (10)  45 (90)  17 (34)  33 (66)  3.5 (1-10)  8 (3-14) | 0.453  0.440  0.888  0.584  0.837  1  0.563  0.824  0.734  0.767  0.651  0.706  0.911  0.437 |

BMI Body Mass Index. ASA American Society of Anesthesiologists Score. CCI Charlson Comorbidity Index. PDAC Pancreatic Ductal Adenocarcinoma. IPMN Intraductal Papillary Mucinous Neoplasm. Statistical significance was set at *p* < 0.05): statistically significant values are underlined.
